# Atrio‐ventricular synchronous pacing with a single chamber leadless pacemaker: Programming and trouble shooting for common clinical scenarios

**DOI:** 10.1111/jce.14807

**Published:** 2020-11-18

**Authors:** Mikhael F. El‐Chami, Neal K. Bhatia, Faisal M. Merchant

**Affiliations:** ^1^ Section of Electrophysiology, Division of Cardiology Emory University School of Medicine Atlanta Georgia USA

**Keywords:** atrial tracking, AV synchrony, Micra, Micra AV

## Abstract

Micra leadless pacemaker has progressed from a single chamber pacemaker that can deliver VVIR pacing to a pacing device that can provide atrio‐ventricular (AV) synchrony via a unique pacing algorithm that relies on identifying mechanical atrial contraction. This novel algorithm has its own limitations and intricacies. In this paper, we review this algorithm, suggest steps for troubleshooting and programming these devices and provide clinical examples of Micra AV cases that required changes in programming for adequate tracking of atrial activity.

## 1 INTRODUCTION

Leadless pacemakers (LP) are an alternative to traditional transvenous pacemakers (TV‐PPM) for the treatment of brady‐arrhythmias.^1^, ^2^, ^3^ The Micra transcatheter pacing system (Medtronic) is currently the only United States Food and Drug Administration (FDA) approved LP. It has evolved from a single chamber VVI‐R pacemaker to a device that can provide atrio‐ventricular (AV) synchrony.^4^ The algorithm used to provide AV synchrony is novel and relies on the ability of a three‐axis accelerometer to detect atrial contraction.^5^ Unlike traditional TV‐PPM which sense atrial electrical activity directly through a lead implanted in the atrium, the Micra AV algorithm identifies mechanical atrial contraction, detected by the device imbedded in the ventricle, and allows for AV synchronous pacing.^4^, ^6^

In this paper, we will review this novel algorithm, identify limitations and describe a simple method for programming and troubleshooting the algorithm in common clinical scenarios.

## 2 MICRA ATRIO‐VENTRICULAR ALGORITHM

The Micra AV algorithm relies on the fact that the Micra accelerometer is able to detect four mechanical signals which correspond to different stages of the cardiac cycle.^5^, ^7^

**Figure 1:**
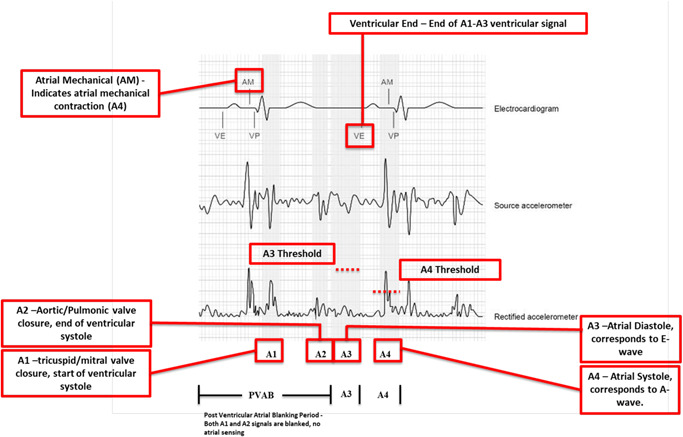
Micra AV accelerometer signals and their relationship to surface ECG wave. AM, atrial mechanical; AV, atrioventricular; ECG, electrocardiography; PVAB, post ventricular atrial blanking; VE, end of ventricular ectopy

The A1 signal (Figure 1) corresponds to tricuspid and mitral valve closure, corresponding to the onset of ventricular isovolumic contraction, and hence falls at the end of the electrocardiographic (ECG) QRS complex (electrical systole precedes mechanical systole). The A2 signal corresponds to aortic and pulmonic valve closures, corresponding to the end of ventricular systole. Hence the A2 signal typically falls at the end of T wave. The A3 signal corresponds to passive ventricular filling while the A4 signal corresponds to atrial contraction. The A3 and A4 signals correspond to the E and A mitral inflow echocardiographic measurements.

The “VE” annotation seen on the Medtronic programmer denotes the end of all ventricular activity (corresponding to mechanical events A1–A3). The A4 is denoted by an “AM” annotation (atrial mechanical), typically occurring around 100 ms after the P wave (corresponding to the electromechanical delay).

Several programmable intervals can be used to troubleshoot and optimize pacemaker function.

(1). Post‐ventricular atrial blanking period (PVAB) used to blank the A1 and A2 signals
(2). A3 detection window (A3 window end)
(3). A3 threshold—typically set to avoid sensing ventricular A3 signals (similar to sensitivity settings in TV‐PPM)
(4). A4 threshold—typically set to ensure atrial A4 sensing (similar to sensitivity settings in TV‐PPM)

## 3 GENERAL RULES TO CONSIDER DURING DEVICE TROUBLESHOOTING

It is imperative to correctly identify intracardiac signals, in particular the A4. In patients with complete heart block (CHB), it can be difficult to identify the appropriate atrial signals. A systematic approach, starting with a noise‐free surface ECG with a clear P wave is crucial. The Lewis lead method can be used in cases where the P wave is not easily discernable on traditional ECG leads.^8^ After identifying a clear ECG P wave, the next step is to correctly line up the A1‐A4 signals under the corresponding ECG signals (Figure 1).

If the A4 signal is small or difficult to identify, the accelerometer atrial‐sensing vector can be changed. Subsequently, the device can run a manual test: the manual atrial mechanical (MAM) test, to get a clear view of the A1–A4 signals in respect to their correlation with the surface ECG. In our experience it is best to first run this test in the ventricle, dual, inhibited mode, which allows for clear distinction of the A1–A4 signals without the device potentially complicating troubleshooting by attempting to track atrial activity. The MAM test can then be run in the ventricle, dual, dual response (VDD) mode, where the clinician can make programming adjustments while observing the device attempt atrial tracking. An additional benefit to running the MAM test in VDD is that it allows the user to identify reasons of failure (i.e., A4 undersensing, etc.) when the device does not track as intended.

During the MAM test it is useful to assess the underlying atrial rate, as this will help determine what the expected ventricular rate should be if the atrial signal is being tracked appropriately. Additionally, when running the MAM test, it is advisable to turn off the “auto” atrial mechanical features (i.e. auto A3 threshold, auto A3 window end, and auto A4 threshold). This prevents the device from autocorrecting and changing in real‐time the programmed intervals that are being used. The “auto” features can be turned back on at the end, once appropriate baseline values are established. In our experience, manual adjustments of the A4 threshold, A3 threshold, and A3 window end are common and often necessary even if the “atrial sensing setup” feature and MAM test are utilized. The atrial sensing set up is an automated feature that checks all three orthogonal accelerometer vectors and choose the one that provides the best A4 signal.

Table 1 summarizes the steps needed for efficient programming and troubleshooting of the Micra AV.

**Table 1:** Suggested troubleshooting steps

**Stepwise approach to troubleshooting Micra AV tracking**
Ensure surface ECG lead has a clear P wave
Correlate A1‐A4 with surface ECG
Adjust/change to a different sensing vector if A4 is of small amplitude
MAM test in VDI mode, then VDD mode
Optimize A4/AM sensing and tracking:
(1). Changing A4 threshold (2). Adjusting A3 window end (3). Changing A3 threshold (4). Adjusting PVAB

Abbreviations: AV, atrioventricular; ECG, electrocardiogram; MAM, manual atrial tracking; PVAB, post ventricular atrial blanking; VDI, ventricle, dual, inhibited; VDD, ventricle, dual, dual response.

## 4 TROUBLESHOOTING EXAMPLES

In this manuscript we will discuss programming and troubleshooting Micra AV implants that we have encountered in our practice.

## 5 CASE 1

A 72‐year‐old female with intermittent high‐grade AV block received a Micra AV.

**Figure 2:**
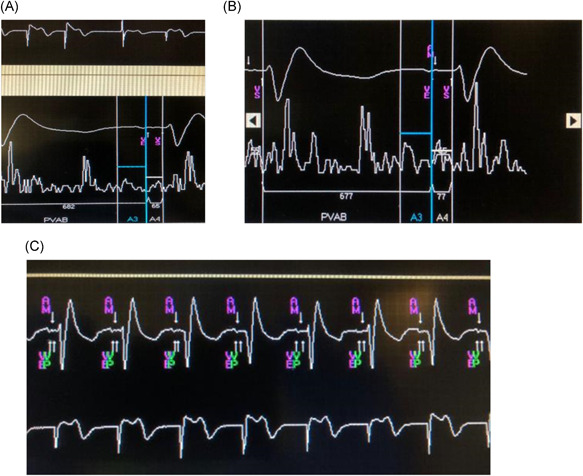
(A) The A4 signal falling below threshold which resulted in undersensing and lack of atrial tracking (see text for detail). (B) Next, A4 threshold was adjusted which resulted in A4 sensing and AM marker on the device channel. (C) AV synchrony established as evident by AM and VP). AM, atrial mechanical; VP, ventricular pacing

Figure 2A shows intermittent AV block with no atrial tracking. A MAM test was performed and the A4 can be seen to occur after the P wave. The A4 is undersensed (amplitude falling below the A4 threshold) which is manifest by the lack of an AM marker. After adjusting the A4 threshold (lowered from 1.2 to 0.7 m/s^2^), adequate sensing of A4 is present (Figure 2B) and AM is appropriately annotated. This simple change results in appropriate atrial tracking with restoration of AV synchrony (Figure 2C).

## 6 CASE 2

**Figure 3:**
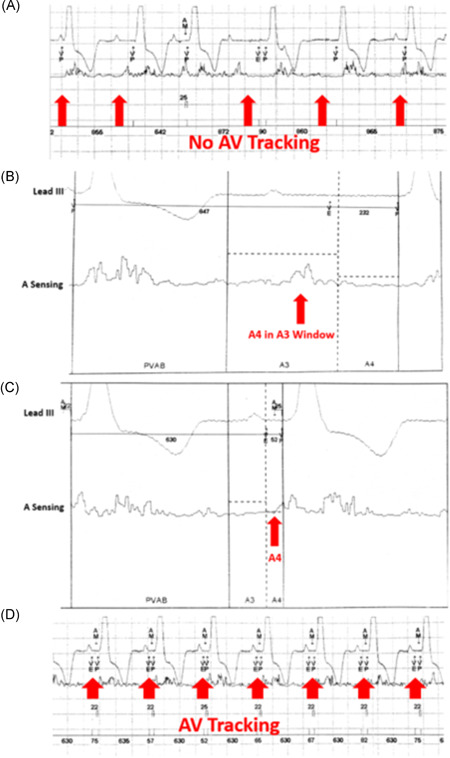
(A) Rhythm strip showing sinus rhythm with complete heart block and intermittent atrial sensing and tracking. (B) Accelerometer signal showing the A4 signal (red arrow) falling in the A3 sensing window, resulting in undersensing. (C) The A3 window end was shortened and A4 (red arrow) is now sensed. (D) AV synchrony established after programming change. AV, atrioventricular

A 78‐year‐old female presented with dizziness and was found to have CHB. She received a Micra AV implant. Her rhythm strip shows AV dissociation with RV pacing and no AV synchrony (Figure 3A). A MAM test was performed and the A4 signal occurs shortly after the surface P wave (Figure 3B). Importantly, the A4 signal is falling in the A3 sensing window, preventing appropriate sensing of the A4 by the device and resulting in lack of AV synchrony. The solution in this situation is to simply shorten the A3 window end interval (970–750 ms) to allow the device to detect the A4 signal. Figure 3C indicates adequate A4 sensing after shortening the A3 sensing window. Additionally, the A4 threshold was increased to avoid sensing noise (1.6–2.0 m/s^2^). It was previously set too low given the lack of a clear A4 signal. These changes resulted in AV synchrony (Figure 3D).

## 7 CASE 3

**Figure 4:**
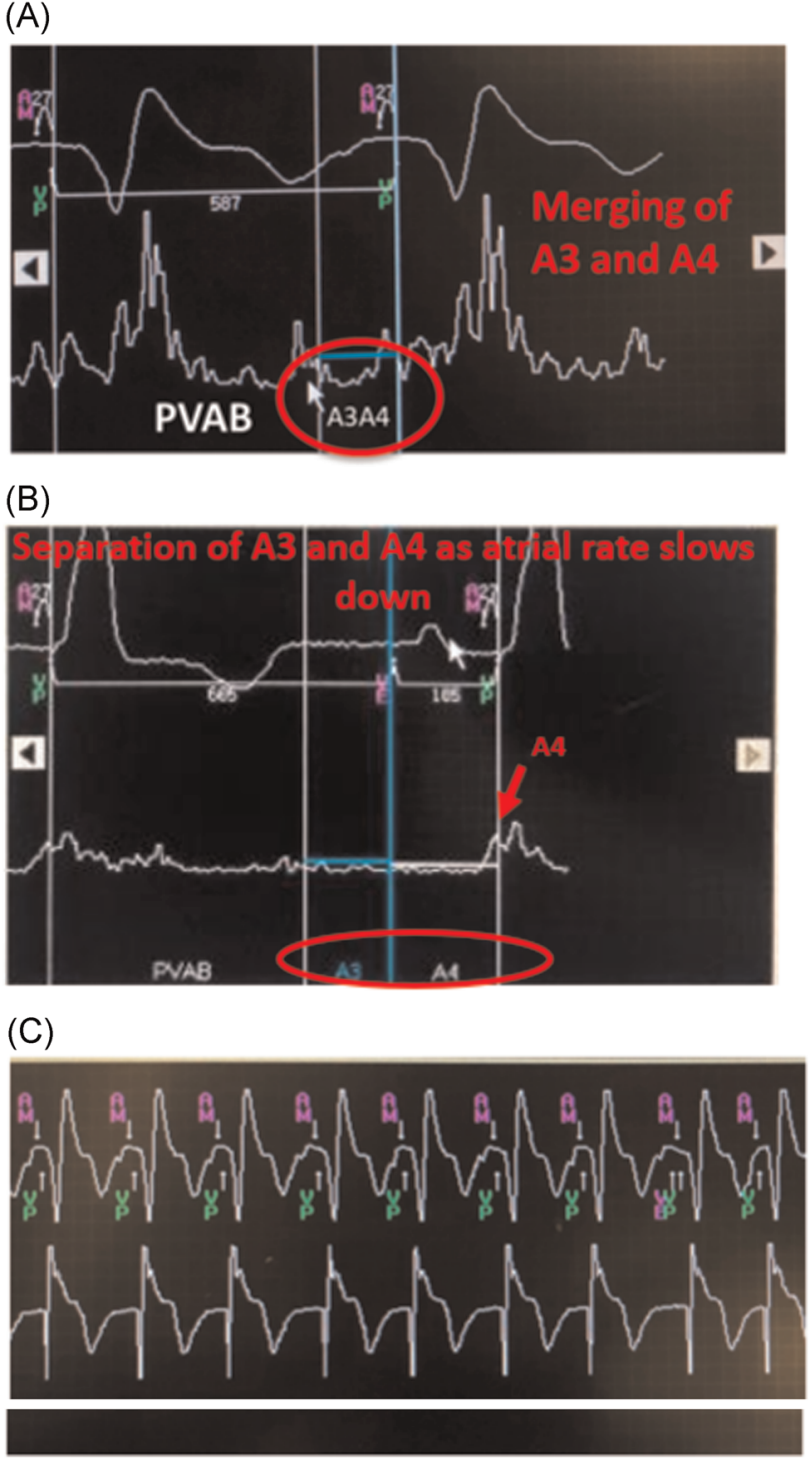
(A) Sinus tachycardia tracked. Accelerometer signals show A3 and A4 merger (A7)(circled). Threshold (horizontal white line) set low enough to detect A4 but high enough to avoid A3. A7 sensing occurs in the A3 window due to the fast atrial rate. (B) Sinus rate slows resulting in the A3 and A4 separating (circled). The auto A3 window end self‐adjusted to keep A3 covered up and A4 (red arrow) tracked. (C) Rhythm strip shows AV synchrony. AV, atrioventricular; PVAB, post ventricular atrial blanking

An 81‐year‐old man presented with CHB. A Micra AV was implanted. The underlying atrial rhythm was sinus tachycardia at a rate of 105 beats per minute (bpm). In cases of relative tachycardia, the A3 and A4 signals can merge, resulting in an “A7” signal (Figure 4A). To ensure appropriate atrial tracking in these scenarios, the A3 threshold has to be lowered sufficiently to detect the A4 signal (A3 and A4 are merged) but kept adequately high enough to remain above the A3 value to avoid inappropriately sensing and tracking A3 signals. In addition, turning the A3 auto threshold function off is essential in this situation because this feature is used to avoid sensing A3 signals by constantly adapting to the A3 threshold. In a scenario such as this case, the A3 auto threshold function may result in undersensing A4 because it is merged with A3. The following day, the sinus rate slowed and A4 sensing remained adequate (Figure 4B). In this situation, the “auto” A3 window end feature remained enabled and the A3 window end was extended as the rate slowed, appropriately masking A3 but allowing A4 to be sensed (Figure 4C).

## 8 CASE 4

A 76‐year‐old female with end stage renal disease on peritoneal disease presented with presyncope and was found to have high‐grade AV block and intermittent CHB.

**Figure 5:**
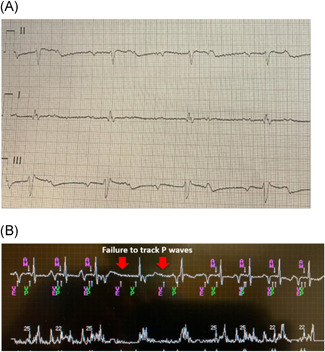
(A) Rhythm strip shows sinus rhythm with complete heart block and junctional escape rhythm. No RV pacing or AV synchrony seen since the device mode switched to VVI + (backup VVI 40) (see text for detail). (B) Mode switch turned off. Partial AV synchrony restored. Some P waves are undersensed due to Wenckebach behavior and some P waves falling in the PVAB period. AV, atrioventricular; ECG, electrocardiography; PVAB, post ventricular atrial blanking; RV, right ventricle; VE, end of ventricular ectopy

A Micra AV implant was performed. The patient was discharged uneventfully but presented again a few days later with dizziness. Her presenting ECG showed CHB with a junctional escape rhythm (JER) at 42 bpm (Figure 5A). Chest radiograph showed stable Micra position. Device interrogation showed sinus rhythm at 80 bpm with CHB and JER. The device was initially programmed VDD with AV Conduction Mode Switch enabled (so called “VVI+”). The Micra AV Conduction Mode Switch algorithm is designed to promote intrinsic conduction when possible. If the ventricular rate is greater than 40 bpm, it switches to VVI 40 regardless of the presence of AV block. This issue presented in this case is also commonly seen in patients with 2:1 AV block when the sinus rate is ≥80 bpm. In these situations, it is often best to turn AV Conduction Mode Switch off to avoid the default switch to back‐up pacing at VVI 40 in the setting of AV block.

In the Micra AV, AV conduction is considered present if the intrinsic ventricular rate exceeds 40 bpm. This algorithm is strictly looking at the intrinsic ventricular rate to assess whether to switch back to VDD. Thus, if you have AV dissociation and a junctional rate of greater than 40 bpm, you will remain in VVI 40 despite AV dissociation. Mode switch to VDD can be observed if ventricular pacing at the lower rate of 40 bpm occurs (2 paced beats out of a rolling window of 4).

Conduction check occurs again after 1 min and if AV block persists, conduction check time is doubled until a maximum time of 8 h.

In this patient with frequent periods of Wenckebach AV conduction, an additional challenge is progressive PR prolongation. As this occurs, the RP shortens and the P wave frequently encroaches on the PVAB. A partial solution in this patient was to both disable the conduction mode switch and also to shorten the PVAB to minimize intermittent P wave blanking. Despite these changes, occasional failure to track P waves continued to occur due to intermittent P wave blanking (Figure 5B).

## 9 DISCUSSION

The Micra AV provides the important benefit of atrial synchronous ventricular pacing in a leadless device but the algorithm has notable limitations. As implantation of these devices is expected to increase, it is important that physicians understand the functionality and specific features of this device.

Patient selection is crucial for a successful outcome with this technology. The algorithm is not designed to track atrial rates greater than 105 bpm. This is due to merging of A3 and A4 and encroachment of A4 on the PVAB as the sinus rate increases. Therefore, the device will switch to VVIR mode, and hence lose atrial synchronous function, at a programmable rate, typically greater than 105 bpm. Hence, in patients with fast baseline sinus rates, younger and more physically active patients, or any patients, irrespective of age, who may have greater reliance on AV synchrony at peak heart rate, this device may not be optimal. In general, the average age of patients with conduction system disease requiring pacing is in the mid 70's.^9^ Most of these elderly patients, in our experience, do quite well with a device that is capable of providing AV synchrony approximately 80% of the time.^5^ Table 2 presents some considerations for device choice in patients with conduction system disease. It is important to weigh the pros and cons of each device and tailor the pacing approach based on patient characteristics and the nature of conduction disease.

**Table 2:** Factors that might influence device choice

**Micra AV >TV‐PPM**	**TV‐PPM >Micra AV**
ESRD	Fast baseline sinus rate
Prior CIED infection	AV synchrony desired with exercise
Avoiding transvenous lead	AV synchrony desired 100%
	Sick sinus syndrome

Abbreviations: AV, atrioventricular; CIED, cardiac implantable electronic device; ESRD, end stage renal disease; TV‐PPM, transvenous permanent pacemaker.

## 10 CONCLUSION

In this manuscript, we review the Micra AV algorithm, identify some if its limitations and propose a simple algorithm for troubleshooting in commonly encountered clinical scenarios.

## CONFLICT OF INTERESTS

Mikhael El‐Chami is a consultant for Medtronic, Boston Scientific and Biotronik. All other authors have no conflict of interests.
